# Development of a universal, oriented antibody immobilization method to functionalize vascular prostheses for enhanced endothelialization for potential clinical application

**DOI:** 10.1186/s13036-023-00356-6

**Published:** 2023-06-01

**Authors:** Qiuwang Zhang, Sebastian Duncan, Daniel A. Szulc, Charles de Mestral, Michael JB Kutryk

**Affiliations:** 1 Division of Cardiology, Keenan Research Center for Biomedical Science, St. Michael’s Hospital, Unity Health Toronto, University of Toronto, Toronto, ON Canada; 2 Division of Vascular Surgery, Keenan Research Center for Biomedical Science, St. Michael’s Hospital, Unity Health Toronto, Toronto, ON Canada; 3 Department of Surgery, Faculty of Medicine, University of Toronto, Toronto, ON Canada

**Keywords:** Cardiovascular prosthetic device, Thrombosis, Endothelial progenitor cells, Oriented antibody immobilization, Universal coating, Clinical application

## Abstract

**Background:**

Thrombosis is a common cause of vascular prosthesis failure. Antibody coating of prostheses to capture circulating endothelial progenitor cells to aid endothelialization on the device surface appears a promising solution to prevent thrombus formation. Compared with random antibody immobilization, oriented antibody coating (OAC) increases antibody-antigen binding capacity and reduces antibody immunogenicity in vivo. Currently, few OAC methods have been documented, with none possessing clinical application potential.

**Results:**

Dopamine and the linker amino-PEG8-hydrazide-t-boc were successfully deposited on the surface of cobalt chromium (CC) discs, CC stents and expanded polytetrafluoroethylene (ePTFE) grafts under a slightly basic condition. CD34 antibodies were immobilized through the reaction between aldehydes in the Fc region created by oxidation and hydrazides in the linker after t-boc removal. CD34 antibody-coated surfaces were integral and smooth as shown by scanning electron microscopy (SEM), had significantly reduced or no substrate-specific signals as revealed by X-ray photoelectron spectroscopy, were hospitable for HUVEC growth as demonstrated by cell proliferation assay, and specifically bound CD34 + cells as shown by cell binding testing. CD34 antibody coating turned hydrophobic property of ePTFE grafts to hydrophilic. In a porcine carotid artery interposition model, a confluent monolayer of cobblestone-shaped CD31 + endothelial cells on the luminal surface of the CD34 antibody coated ePTFE graft were observed. In contrast, thrombi and fibrin fibers on the bare graft, and sporadic cells on the graft coated by chemicals without antibodies were seen.

**Conclusion:**

A universal, OAC method was developed. Our in vitro and in vivo data suggest that the method can be potentially translated into clinical application, e.g., modifying ePTFE grafts to mitigate their thrombotic propensity and possibly provide for improved long-term patency for small-diameter grafts.

**Supplementary Information:**

The online version contains supplementary material available at 10.1186/s13036-023-00356-6.

## Introduction

Lifestyle modification, pharmacotherapy and surgical interventions have reduced the incidence, and improved the outcome, of atherosclerotic vascular disease. When necessary, endovascular procedures such as balloon angioplasty, the insertion of metallic stents made of metal alloys such as cobalt chromium (CC), or atherectomy can be performed to widen a stenosed vessel. Alternatively, an autologous vein graft or a synthetic conduit made from non-degradable polymers such as expanded polytetrafluoroethylene (ePTFE) can be used to accomplish arterial revascularization. ePTFE is also used for arteriovenous (AV) grafts for hemodialysis.

Intermediate and large diameter ePTFE grafts have shown success in clinical applications such as aortic and aortoiliac reconstructions [1]. In contrast, small caliber conduits, typically required for infrainguinal bypass (≤ 6 mm), coronary artery bypass (5-6 mm), or AV grafts for hemodialysis (6-8 mm), show poor long term patency, attributed to a more demanding hemodynamic environment and thrombus formation. The five year patency rate of femoral-popliteal bypass with prosthetic grafts is reported to be between 40 to 60% while the primary patency of AV grafts for hemodialysis is 28% at 2 years [2, 3]. Early clinical trials of ePTFE grafts for coronary artery bypass showed a patency rate ranging between 60% at 12 months and 14% at 45 months [4, 5]. To mitigate pro-thrombotic property of ePTFE grafts for improved long-term patency, seeding of autologous endothelial cells on the graft for coronary bypass was explored by Dohmen et al., who demonstrated a patency rate of 50% at 72 months [6]. This technique, however, makes the manufacturing of off-the-shelf products infeasible. Currently, there are no approved ePTFE grafts for coronary artery bypass.

Since the first descriptions of endothelial progenitor cells (EPCs), a subset of CD34 + mononuclear cells circulating in the blood [7], and the finding that the main source of the endothelial cells lining the prosthetic implant are bone marrow derived [8], extensive studies have explored antibody coating of intravascular devices to capture circulating EPCs for enhanced endothelialization [9–11]. The COMBO® Dual Therapy Drug Eluting Stent (OrbusNeich Medical Technologies) is configured with a luminal polymeric dextran coating with embedded mouse monoclonal anti-human CD34 antibodies to capture EPCs to enhance the natural endothelialization process [12]. Although the dextran coating technology has proven effective for CD34 + cell capture on metallic stents, it is not as effective for the capture of EPCs when coated on surfaces such as ePTFE [13–15]. Coating strategies dependent on nonspecific physical adsorption, covalent conjugation, and matrix adhesion result in random orientation of antibodies, where antibodies are immobilized in two manners: some attach to the substrate through the Fab fragment leaving the antigen binding region inaccessible to antigen, while the others through the Fc fragment leaving the Fab region free for antigen binding [13–24]. In contrast, oriented antibody immobilization relies solely on the attachment of the Fc fragment on the substrate, leaving all Fab fragments free for antigen binding [25]. An oriented antibody immobilization method suitable for functionalization of multiple substrates for potential clinical application is needed but has not been established.

Dopamine, through oxidative self-polymerization, can be deposited onto a wide variety of materials [26]. The polymerized dopamine film is an extremely versatile platform for secondary reactions, and has been used to immobilize thiolated polyethylene glycol (PEG), aminated-PEG, trypsin, peptide, bovine serum albumin, concanavalin A and RNase B [27, 28]. The aim of this study was to develop a means for oriented antibody immobilization utilizing dopamine polymerization as the base of the universal coating and polyethylene glycol (PEG) as the linker for antibody orientation. The effectiveness of the coating was tested in vitro on electropolished CC discs, CC stents and ePTFE meterial, as well as in vivo on ePTFE grafts with a diameter of 5 mm using a pig carotid interposition model.

## Results

### Oriented antibody coating

**Fig. 1:**
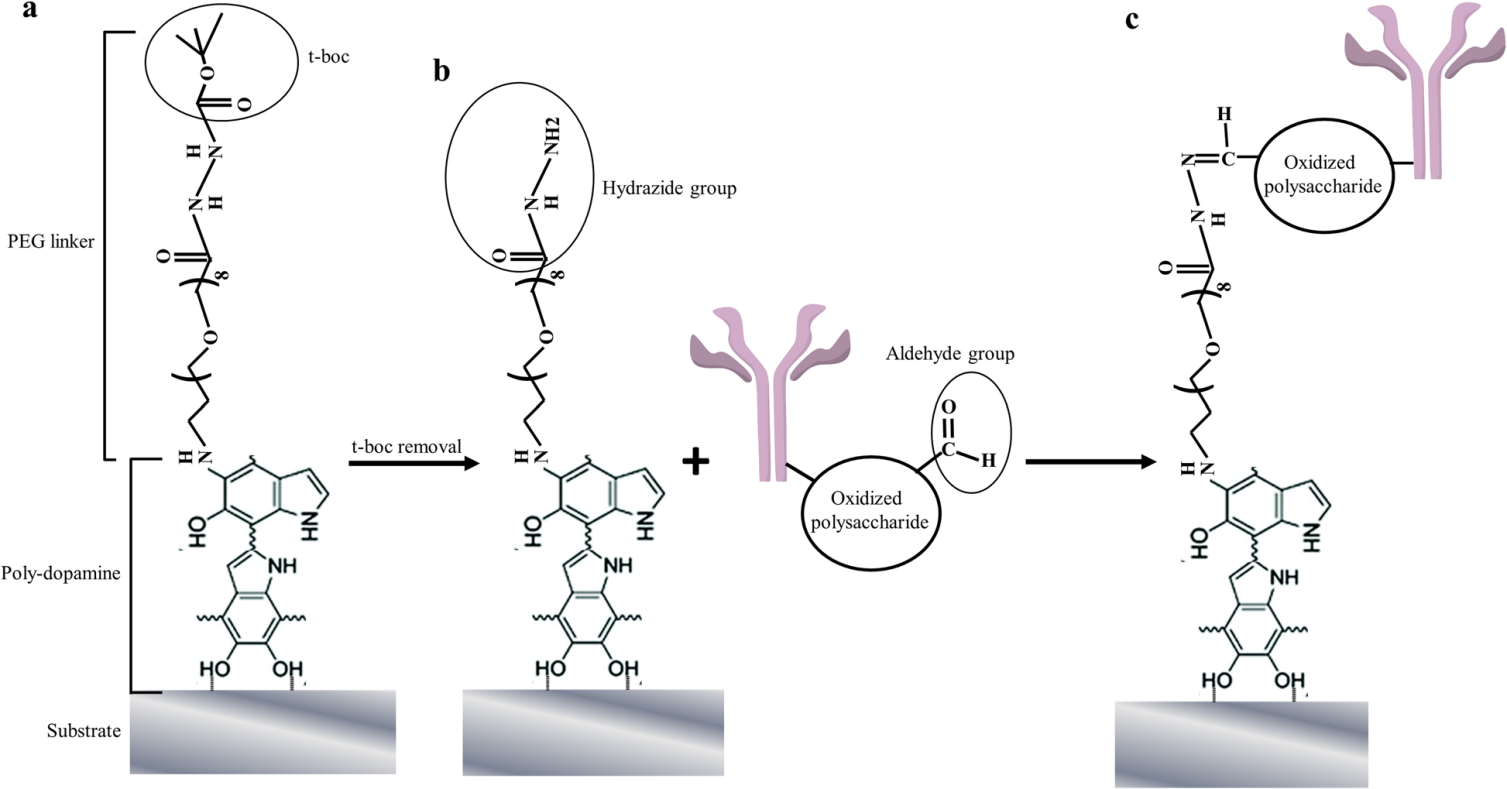
Schematic diagram of the coating procedure. The ability of dopamine to deposit on multiple substrates through self-polymerization is the foundation of the universality of the coating method developed in this study (panel **a**). After removal of the protective t-boc group, hydrazides in the linker react with aldehydes created by oxidation of cis-diols in the polysaccharide moieties of Fc region (panel **b**), resulting in oriented antibody coating (panel **c**)

Figure 1 shows the schematic diagram demonstrating the mechanism and process of the universal, oriented antibody coating method developed for this study. The foundation of the universality relies on the ability of dopamine to deposit on virtually any substrate through self-polymerization. Amino-(PEG)_8_-hydrazide-t-boc as the linker is attached to the poly-dopamine layer through the reaction between the hydroxyl group from dopamine and the amine group from the linker (Fig. 1a). After removal of the protective t-boc group, hydrazides in the linker are exposed, and react with aldehydes created by oxidation of cis-diols in the polysaccharide moieties of Fc region (Fig. 1b), resulting in oriented antibody attachment that makes the Fab fragment accessible to antigen-binding (Fig. 1c).

### Characterization of coated materials

#### SEM analysis

**Fig. 2:**
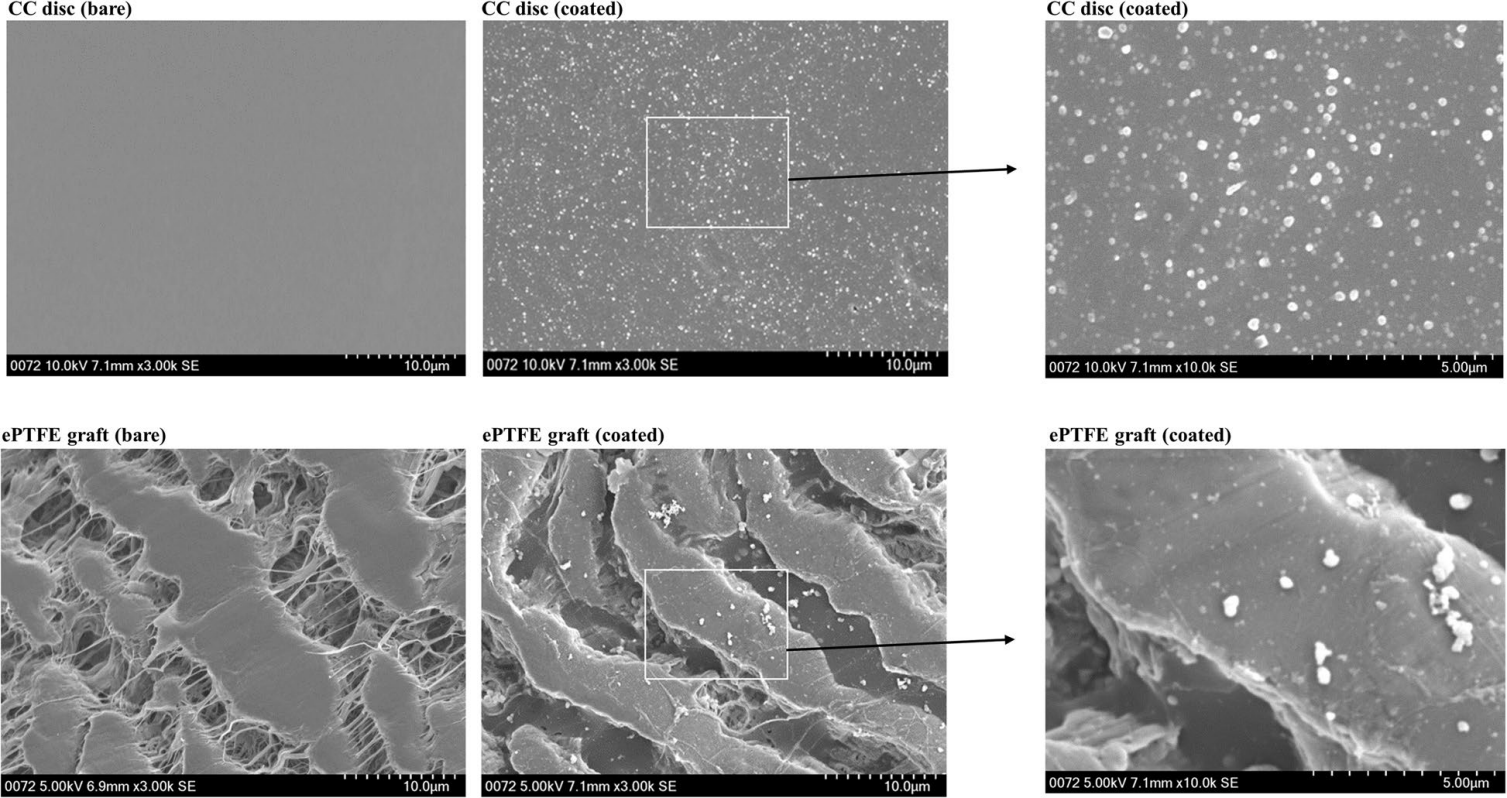
SEM examination of the coating surface. Integral and smooth coating was seen on both CC discs and ePTFE grafts as indicated. Dopamine aggregates can be seen on the coated surface, with most having a size of < 500 nm

As shown in Fig. 2, the coating surface is integral and smooth. Polydopamine aggregates were seen on both discs and grafts with a maximum size of approximately 500 nm (Fig. 2).

#### Mechanical characterization

**Fig. 3:**
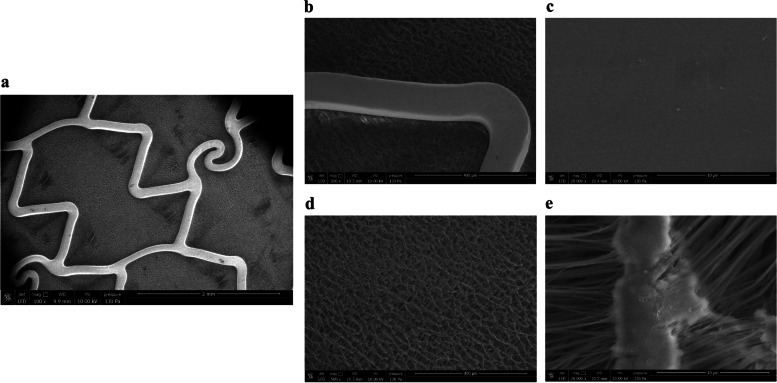
Assessment of the integrity and deformability of the coating on an expanded Graftmaster RX coronary stent system by SEM. Panel **a** shows a coated Graftmaster RX coronary stent system before expansion. After expansion, no cracking, fissuring, or peeling was seen on the coated metal surface (panels **b** and **c**), and on the ePTFE the coating remained elastic with no peeling or flaking (panels **d** and **e**)

With expansion, the coating on the stent struts remained smooth and confluent with no cracking, fissuring, or peeling (Fig. 3). On the ePTFE of the Graftmaster system the coating remained elastic with no peeling or flaking (Fig. 3).

#### Immunofluorescence analysis

Green fluorescence (FITC) was observed on CC discs coated with CD34 antibody (Supplementary Fig. 1a) but not isotype control (Supplementary Fig. 1b), suggesting a specific interaction between the coated CD34 antibody and CD34 peptide.

#### XPS analysis

**Fig. 4:**
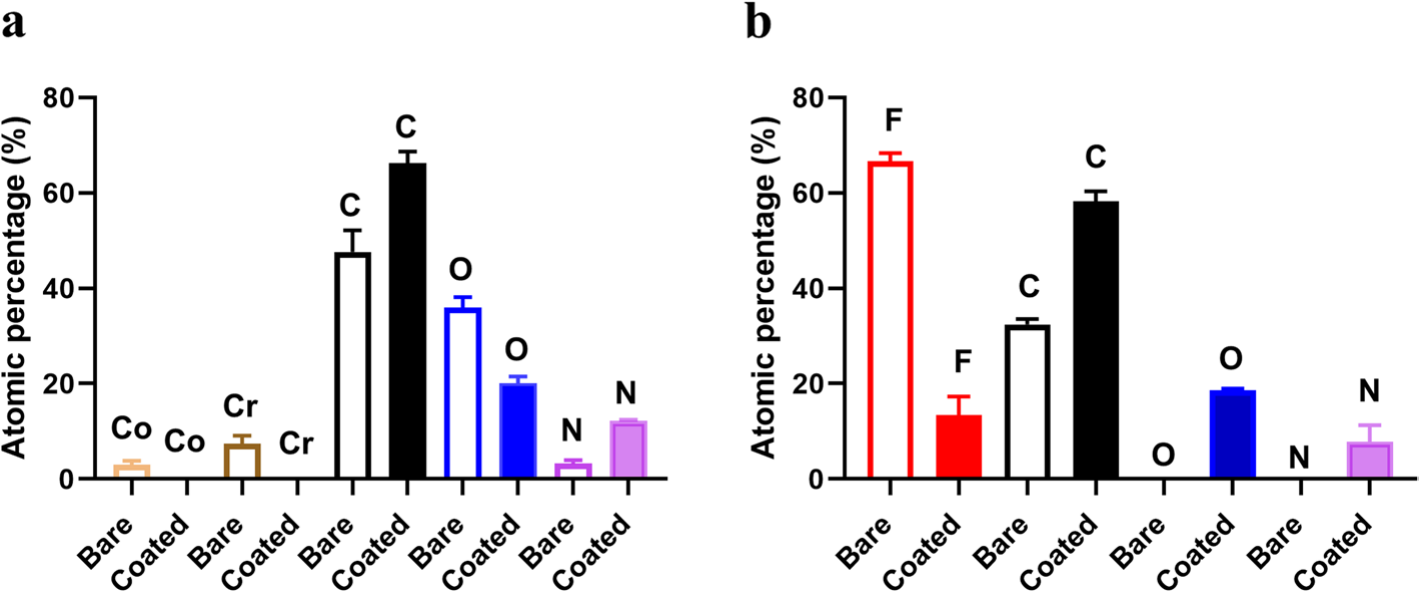
XPS analysis results. As shown in panel **a**, cobalt and chromium were not detectable on the coated disc surface, indicating confluent coverage by the coating. Panel **b** shows the result of coated grafts: carbon and fluorine account for > 99% of chemical elements in bare ePTFE grafts; and coating significantly reduced fluorine percentage. The residual fluorine in the coated ePTFE graft detected by XPS (panel **b**) is likely from internodal spaces where insufficient coating may occur. Co: cobalt; Cr: chromium; F: fluorine; C: carbon; O: oxygen; and N: nitrogen

XPS analysis revealed the absence of cobalt and chromium signals from coated CC discs (Fig. 4a, *n* = 3). Carbon and fluorine were the major chemical elements of bare ePTFE grafts accounting for 32.4 ± 1.1% and 66.6 ± 1.8%, respectively (Fig. 4b), and the percentage of fluorine dropped significantly to 13.3 ± 3.9% (*p* = 0.005, *n* = 3) in coated ePTFE grafts (Fig. 4b). Representative spectra for bare and CD34 antibody coated ePTFE material, and of a bare and a coated disc were shown in Supplementary Fig. 2.

#### Contact angle measurement

**Fig. 5:**
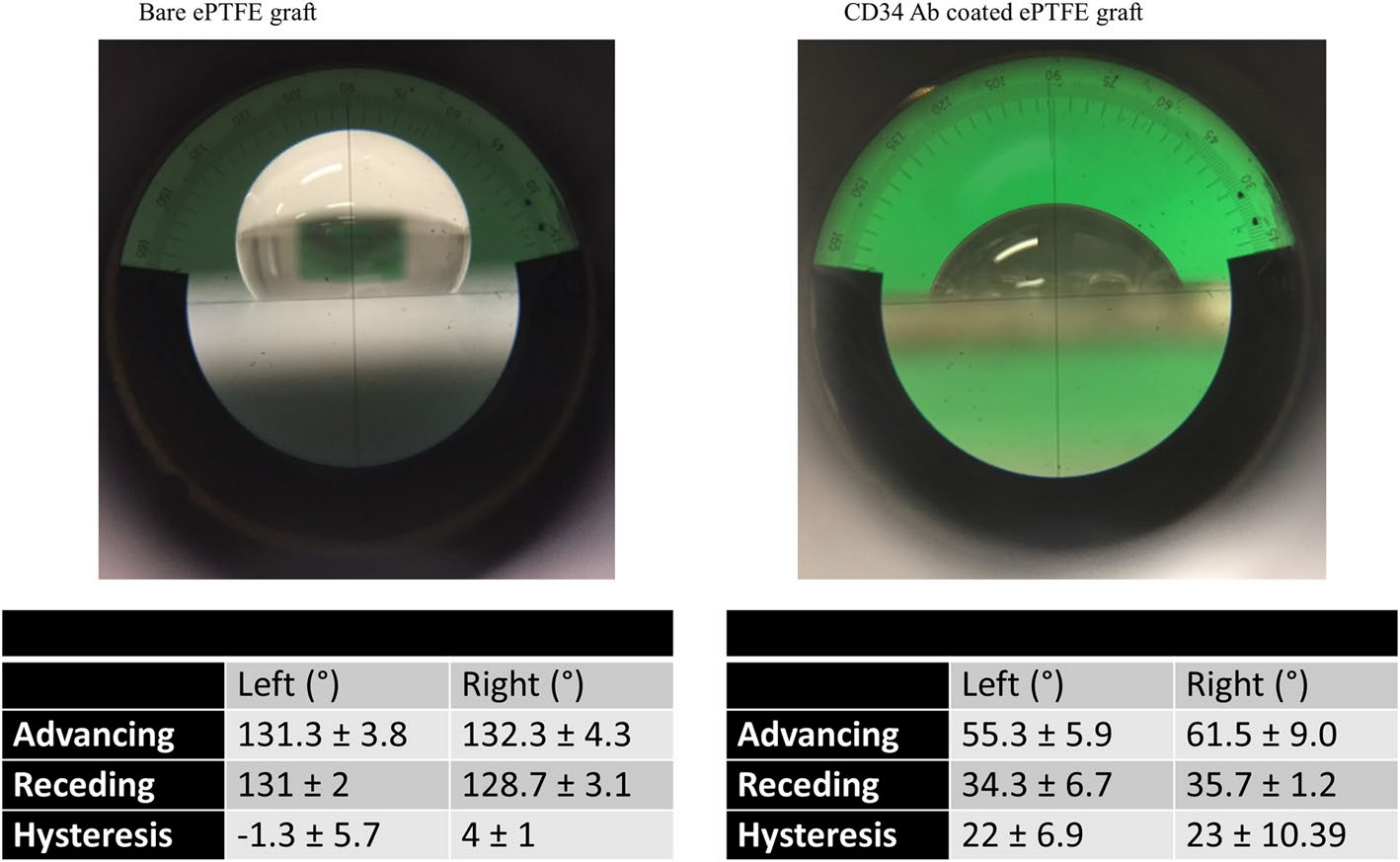
Contact angle measurement. Shown in this figure are the results of bare and coated ePTFE grafts. Coating substantially reduced advancing and receding angles while increased hysteresis, indicating that coating resulted in high hydrophilicity and low hydrophobicity

Coating of ePTFE material markedly reduced the left and right advancing angles (*p* = 0.0001 and *p* = 0.001, respectively) and the left and right receding angles (*p* = 0.0007 and *p* < 0.0001, respectively) (Fig. 5, *n* = 3).

#### Cell growth assessment

**Fig. 6:**
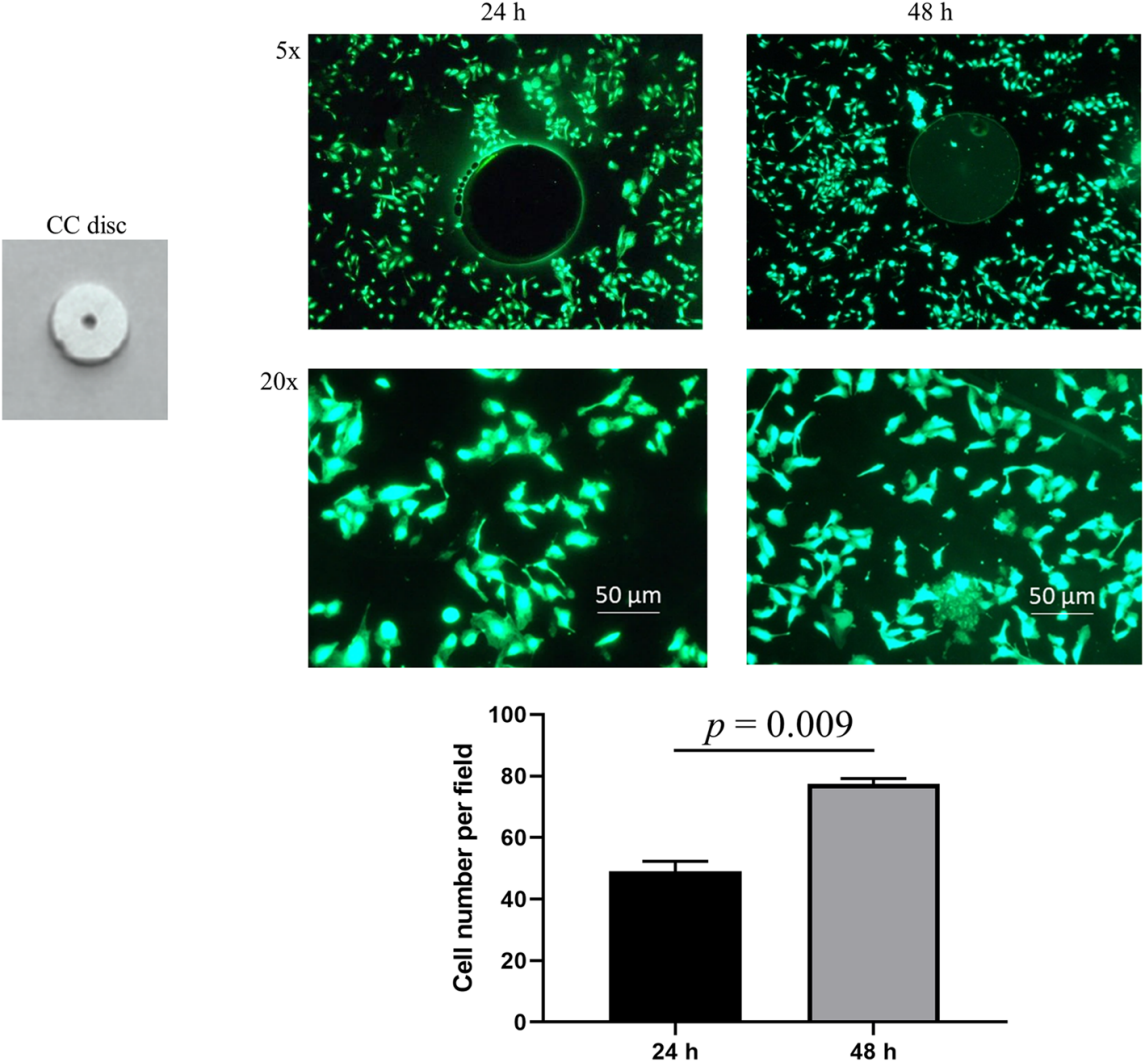
Cell growth assessment. A CC-disc is shown at the top left. HUVECs grown on CD34 antibody coated discs at 24 h and 48 h are shown as indicated. Live cell staining was done using the fluorescent dye Calcein-AM. There was a significantly higher number of live cells grown on the coated disc at 48 h than at 24 h (bottom graph, and cells were counted under 20 × magnification)

HUVECs were shown to grow robustly on the coated disc (Fig. 6) with the cell number of 77.5 ± 1.7/field at 48 h, significantly higher than 49.0 ± 3.3/field at 24 h (*p* = 0.009, *n* = 3).

#### Cell binding testing

**Fig. 7:**
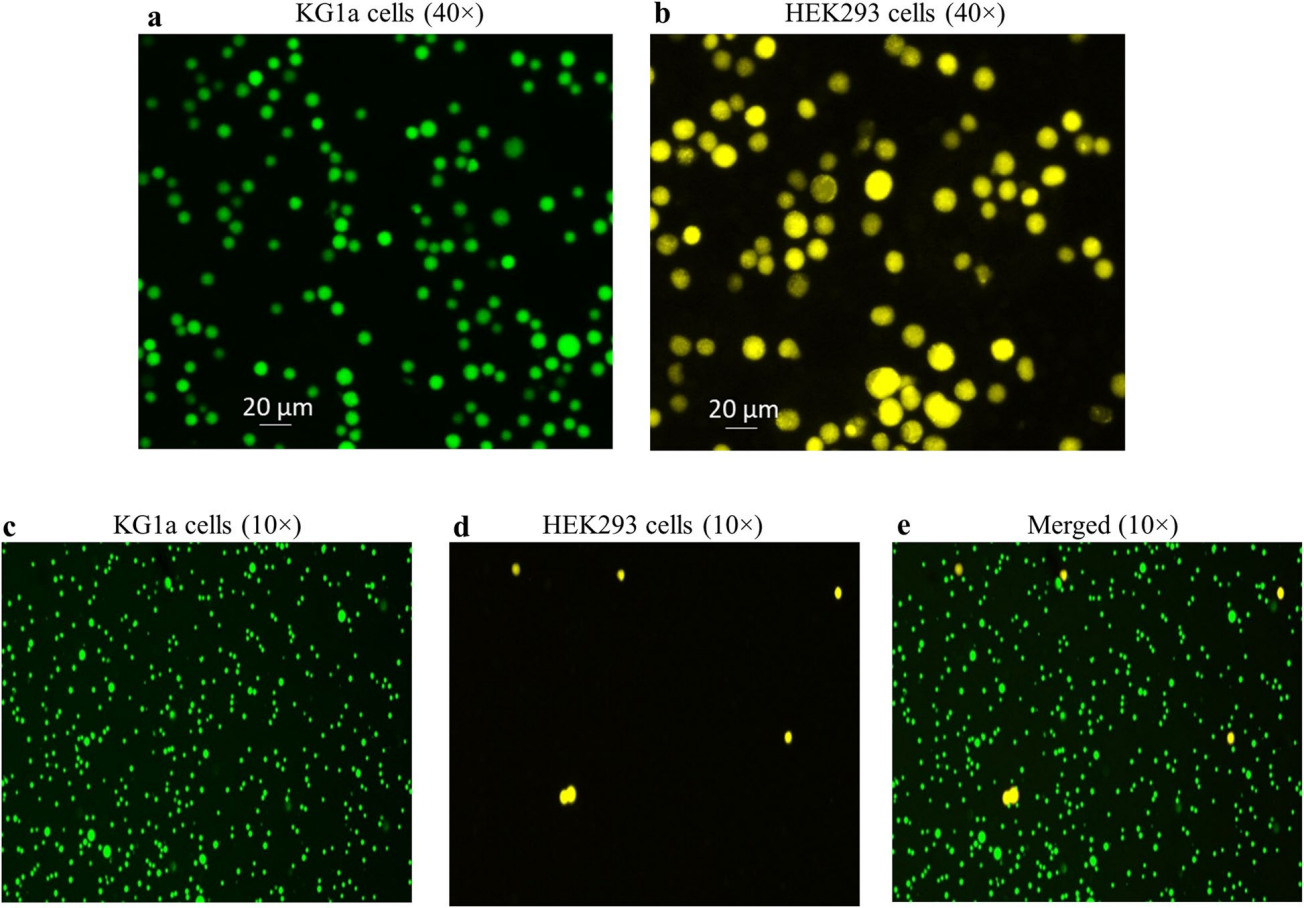
Cell binding testing using mixed pre-stained KG1a and HEK293 cells. Calcein-AM stained KG1a cells (green) and CMTMR stained HEK293 cells (yellow) are shown in panels **a** and **b**, respectively. CD34 antibody-coated substrates were incubated with the mixed pre-stained KG1a and HEK293 cells, and as shown in panel **c**, numerous KG1a cells bound the substrate while HEK293 cells barely bound (panel **d**). Penal e shows the merged image

**Fig. 8:**
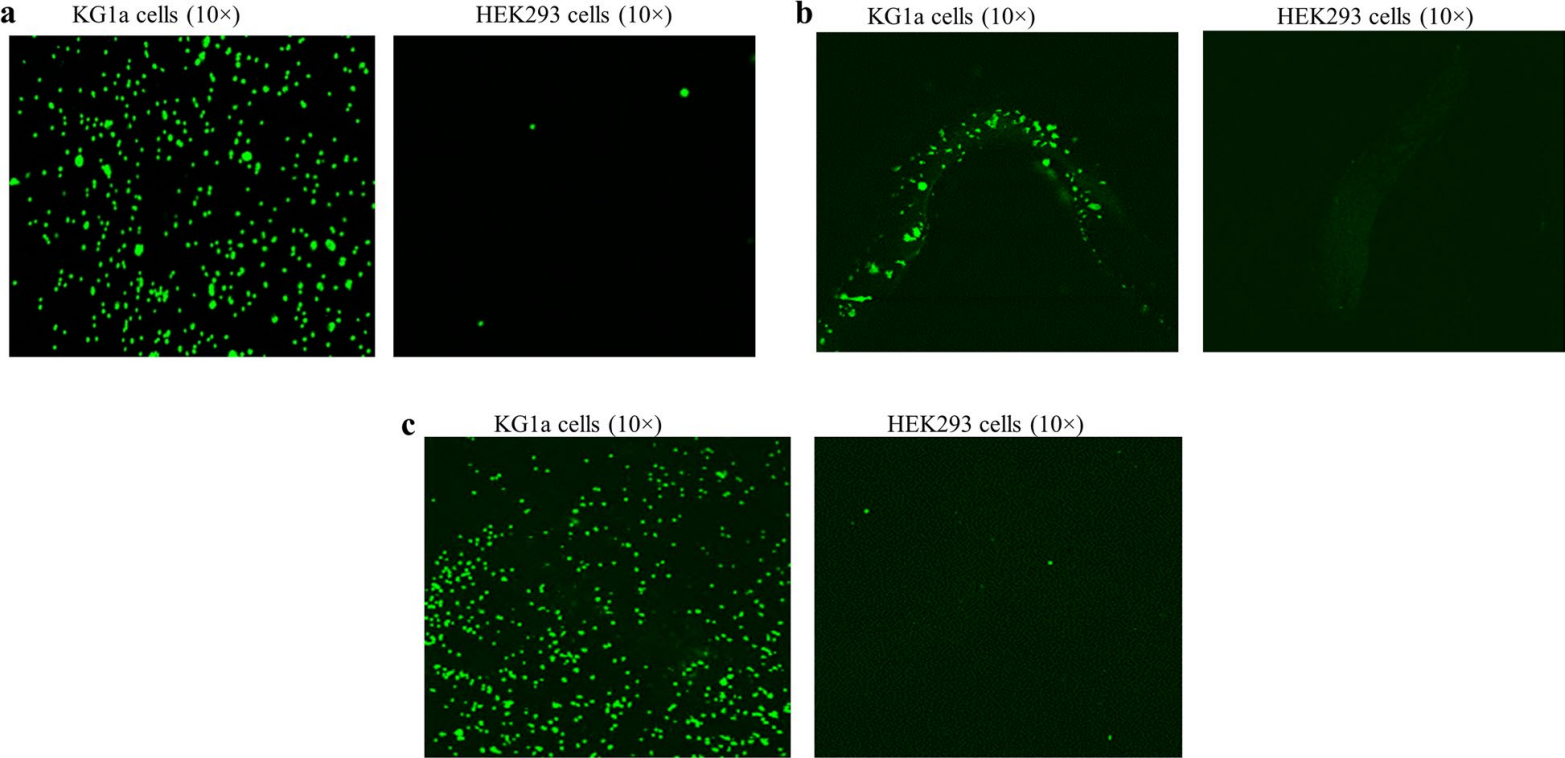
Cell binding analysis. CD34 antibody coated materials were incubated with KG1a or HEK293 cells, and the bound cells were demonstrated by nuclear staining with fluorescence dye Sytox Green. As shown in this figure, all CD34 antibody-coated materials bound CD34 + KG1a cells but not CD34- HEK293 cells as indicated. **a** coated CC disc; **b** coated CC stent; **c** coated ePTFE graft

Fluorescence stained KG1a and HEK293 cells are shown in panels a and b respectively in Fig. 7. Cell binding analysis using mixed pre-stained KG1a and HEK293 cells revealed that numerous KG1a cells (panel c) but few HEK293 cells (panel d) bound the CD34 antibody coated substrate (Fig. 7, panel e shows the merged image). In a separate cell binding assay, it was demonstrated that CD34 antibody coated substrates (CC discs, CC stents, ePTFE grafts) bound CD34 + KG1a cells but not CD34- HEK293 cells (Fig. 8). Additionally, isotype control coated and intermediately coated substrates, and substrates coated without t-boc removal did not bind KG1a cells (Supplementary Fig. 3). Stability analysis demonstrated that after stored in the SB for 2 weeks,** t**he CD34 antibody coated substrate robustly bound KG1a cells but did not bind HEK293 cells, with a similar cell binding pattern seen in Fig. 8 (data not shown).

### In vivo testing

**Fig. 9:**
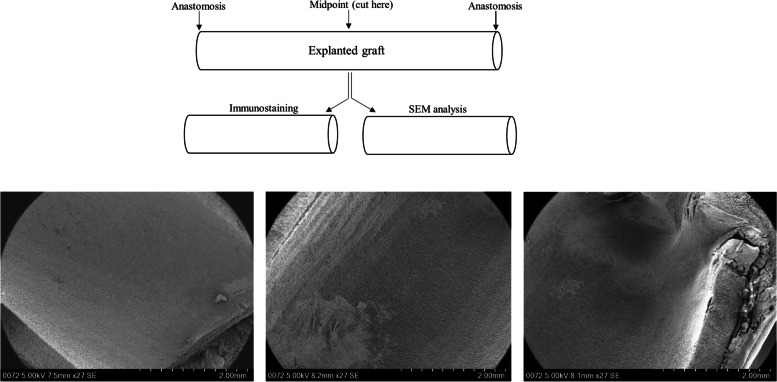
Explanted graft analysis. The upper panel shows a schematic diagram of the analytic process. The lower panels show the longitudinal sections of an explanted CD34 antibody coated graft analyzed by SEM, which revealed near complete endothelialization in every section of the graft

**Fig. 10:**
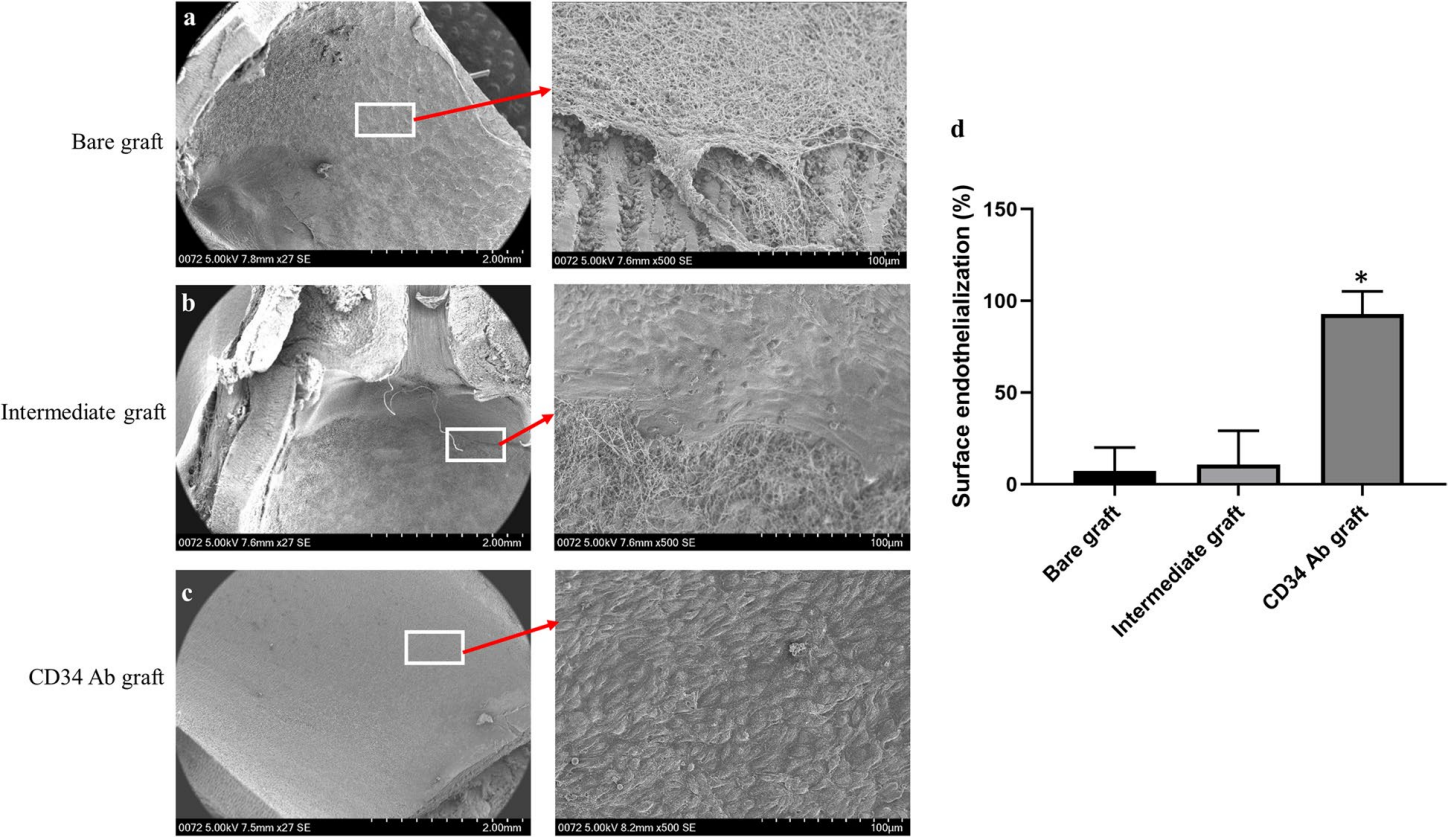
SEM analysis of ePTFE grafts explanted from pigs. While endothelialization was hardly seen in bare and intermediately coated grafts (representative images are shown in panels **a** and **b**, respectively), near complete endothelialization occurred in CD34 antibody (Ab) coated grafts (a representative image is shown in panel **c**). Of note, thrombi (most likely fibrin fiber structures) formed on the luminal wall of bare grafts were observed (panel **a**). Graph d shows the quantification results of endothelialization (*n* = 3 for each group of explants). A significantly higher endothelialization was achieved in the CD34 Ab coated grafts. * *p* = 0.001 compared with bare or intermediately coated grafts

**Fig. 11:**
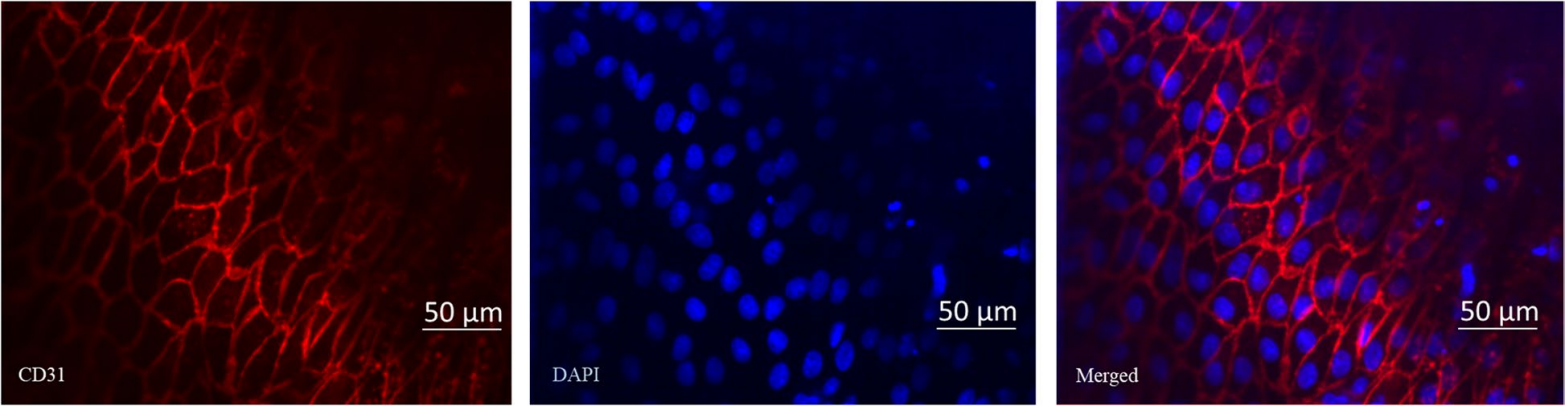
Immunostaining of CD31. CD31 immunostaining of an explanted CD34 antibody graft is shown in this figure. Cells covering the luminal wall of CD34 antibody grafts were stained positive for CD31 (red), indicating endothelial linage. DAPI nuclear staining appeared blue

The ability of CD34 antibody coated ePTFE grafts to capture circulating EPCs for enhanced endothelialization was assessed in pigs using the carotid artery graft interposition procedure. A schematic diagram demonstrating the analyses of grafts with SEM and immunostaining, and SEM images of longitudinal sections of an explanted CD34 antibody graft are shown in Fig. 9. Under SEM, thrombi (most likely fibrin fiber structures) formed on the luminal wall were commonly seen for bare grafts (*n* = 3, Fig. 10a) while such phenomena were not observed for the intermediately coated grafts (*n* = 3, Fig. 10b). There was poor endothelialization on both bare and intermediately coated grafts. In contrast, near complete endothelialization was achieved for CD34 antibody coated grafts (*p* = 0.001 compared with bare or intermediately coated grafts, *n* = 3, panels c and d of Fig. 10). As shown in Fig. 11, cells covering the luminal wall of CD34 antibody grafts were positive for CD31, indicative of endothelial cell lineage.

## Discussion

In the present study, we describe a universal, oriented antibody immobilization method which was successfully applied to functionalize CC discs, CC bare stents, and ePTFE grafts with anti-human CD34 antibodies. SEM examination showed integral and smooth coated surfaces. XPS analysis revealed absent or reduced substrate signals from the coated surfaces. In vitro cell binding experiments demonstrated that CD34 antibody coated materials bound CD34 + but not CD34- cells. Bare or intermediately coated surfaces did not bind CD34 + cells. In a pig carotid artery graft interposition model, near complete endothelialization of luminal wall was observed in CD34 antibody grafts while endothelialization was poor in bare or intermediately coated grafts.

The CD34 antibody coated stent (Genous and Combo), when compared with the drug-eluting stent, did not reduce restenosis as shown in clinical studies. The early trial of the Genous device compared with the paclitaxel-eluting Taxus stent revealed a non-significant increase in target vessel failure in the Genous group at one year follow-up [29]; however, at two year follow-up there was a convergence of the target vessel failure curves of the two groups [30]; of note, no stent thrombosis was observed in the Genous stent group at both follow-ups, compared to 4 and 5 stent thromboses in the Taxus stent group at one year follow up and two year follow up, respectively. The Healing II trial demonstrated that the Genous stent significantly promoted late regression of neointimal hyperplasia, again without acute or late stent thrombosis [31]. More recently, using a longitudinal sequential optical coherence tomography protocol Lee et al. not only showed early robust endothelialization of stent struts, but also late neoimtimal regression without thrombosis with the implantation of Combo (EPC capture/sirolimus eliuting) stents [32]. Overall, the clinical data suggest that although the CD34 antibody stent did not improve target lesion revascularization compared with drug-eluting stents, it induced rapid endothelialization, was of very low thrombotic risk, and found a niche application, i.e., use in patients allergic or intolerant to antiplatelet therapy. The major limitation of the use of small diameter PTFE grafts is their high thrombotic risk. To date, there has been no convincing evidence of a means to significantly accelerate endothelialization to mitigate the thrombotic property of PTFE grafts. Therefore, we aimed to develop such a method by taking into account the finding that CD34 antibody stent promotes endothelialization with reduced thrombosis [29–32].

Many random [13–24], but few oriented antibody coating methods have been reported in the literature [25, 33, 34]. Random antibody attachment not only reduces antibody capability to capture ligands, but also increases the potential for antibody immunogenicity on implantation of the device. Two studies reported processes for oriented antibody coating that rely on purified staphylococcal protein A (SpA) for antibody binding, making the clinical application unlikely [25, 33]. A recent study described oriented antibody coating mediated by a short Fc-binding (RRGW) peptide [34]. RRGW was deposited by chemical conjugation followed by antibody immobilization. Of note, only a polystyrene slide (2 cm × 3 cm) was tested with this method, and a PTFE tape was used around the slide to contain chemical solutions for coating [34]. Apparently, this coating method is not applicable to ePTFE grafts, and its applicability to other materials is also unknown. We sought to develop a clinically relevant method for universal, oriented antibody immobilization. To establish the universality of the coating approach, dopamine was used as the foundation as it can form a polymer film on virtually any substrate through polymerization under a slightly basic condition [26–28]. By simply immersing the substrate in dopamine solution (2 mg/mL in 10 mM Tris–HCl, pH 8.5) for 24 h, Lee et al. showed successful deposition of a poly-dopamine layer on a variety of materials such as stainless steel, glass, quartz, PTFE, polyurethanes, etc., and substrate signals were not detectable after coating [26]. We adopted the same period of incubation time as Lee et al. for deposition of dopamine and the linker on CC discs, CC stents, and ePTFE at 37 °C. As glycosylation of antibodies occurs primarily in the Fc region, the aldehyde group created by oxidation of polysaccharide moieties in antibodies that react with the hydrazide group from the linker results in oriented antibody attachment to the poly-dopamine/PEG layer. We showed that the coating is integral and smooth, in line with previous reports [26, 35–38]. While the percentage of cobalt, chromium and other elements on bare CC discs shown by XPS analysis was in accordance with that reported by others [39], cobalt and chromium signals were not detectable in the coated CC discs. The percentage of fluorine, a major component of ePTFE, was substantially reduced after coating. The residual fluorine signal from the coated surface likely arose from the ePTFE internodal spaces where depression contours can make a complete coating difficult. Contact angle measurement showed the coated grafts to be hydrophilic, compared with the high hydrophobicity of bare ePTFE. Taken together, CD34 antibody coating provides a thin, hydrophilic, and textured surface that is hospitable for EPC binding and proliferation.

The thickness of our coating was measured at approximately 300 nm (data not shown), greater than the 50-130 nm reported previously [26, 37]. As we deposited dopamine at 37 °C in contrast with room temperature adopted by others [26, 37], we speculate that the higher incubation temperature was responsible for the thicker coating. We observed dopamine aggregates on the coated surfaces with the majority < 200 nm and a few approximately 500–1000 nm which fall in the range of size described in the literature [35, 36]. Cell growth assessment revealed that HUVECs grew robustly on the coated CC discs. In previous studies, dopamine coating was shown to promote proliferation of several different cell types including human megakaryocytic cells [26], HUVECs [36], mouse embryonic fibroblasts [37], and human osteoblastic cells [38].

Polydopamine is rich in catechol and amine groups that are responsible for its attachment to virtually any kind of substrates including ePTFE [40, 41]. In vitro studies have examined leaching of polydopamine from coated substrates stored in PBS for up to 5 days, and no leaching was found [42, 43]. However, in vivo leaching has not been described. The potential for in vivo depolymerization, and an adverse downstream effect of coated polydopamine, has yet to be explored.

We showed that CD34 antibody coated substrates robustly bound CD34 + cells while intermediately coated and isotype coated objects did not, and none of these coated substrates bound CD34- cells. These data indicate that immobilized CD34 antibody specifically recognizes CD34 antigen, which is further supported by the immunofluorescence analysis of the immobilized CD34 antibody using a human CD34 peptide (Supplementary Fig. 1). The t-boc protecting group prevents the hydrazide group in the PEG linker from participating in a chemical reaction. Indeed we found that substrates coated with CD34 antibody without t-boc removal did not bind KG1a cells, suggesting the hydrazide group is necessary for antibody immobilization by reacting with the aldehyde group created in the Fc region. This also proves the oriented antibody coating.

A number of studies have investigated antibody coated stents for their ability to promote natural endothelialization in rabbits and pigs [11, 16, 20–22]. In contrast, few studies assessed antibody coated grafts in an animal model [13, 14, 17]. By physical adsorption of polyethylenimine on ePTFE grafts followed by sequential adhesion of heparin, collagen and anti-CD133 antibodies, Lu et al. demonstrated that CD133 antibody coated grafts had remarkably increased endothelialization compared with bare grafts in a porcine carotid artery transplantation model [17]. Of interest, SEM analysis revealed that almost all the cells covering the luminal wall of CD133 antibody coated grafts were spherical, more consistent with the shape of leukocytes rather than the cobblestone appearance of a confluent layer of endothelial cells [17]. In another study, CD34 antibody coated ePTFE grafts using a proprietary process were reported to improve endothelialization, but also to stimulate intimal hyperplasia in a porcine arteriovenous graft model [14]. SEM analysis at an early timepoint (2 weeks) showed a heterogeneous mix of cells, and at a later timepoint (4 weeks) showed a thin, plate-like appearance of the cellular layer, with associated platelets, not typical for healthy endothelium [14]. We tested ePTFE grafts using the same porcine model as Lu et al. [17], and showed near complete endothelialization with cobblestone shaped cells in CD34 antibody coated grafts. Furthermore, we found that cells on CD34 antibody grafts were CD31 positive, indicating endothelial lineage. We chose to do CD31 immunostaining for the assessment of endothelial permeability, as CD31 is expressed at endothelial cell-cell junctions, where it functions to maintain endothelial cell junctional integrity and restore the vascular permeability barrier following adverse challenges [44]. There are other important endothelial junction proteins, such as VE-cadherin, claudin, and occludin that were not analyzed. This is a limitation of the present study. As endothelialization occurs in antibody-coated ePTFE grafts as early as 3 days and nearly completes one week post graft implantation in pigs [14, 17], we did the endpoint study at 2 weeks post graft implantation, leaving long-term patency and intimal hyperplasia unexplored. This is another limitation of this study. Marked intimal hyperplasia is usually detectable two weeks after implantation of antibody coated grafts or after artery injury [14, 45]. Nevertheless, we observed near complete endothelialization in CD34 antibody coated grafts 2 weeks post implantation, suggestive of long-term graft patency and limitation of intimal hyperplasia development.

Reduced circulating EPCs in patients may be a concern for the use of CD34 antibody devices. Nevertheless, there is no data available to suggest that endothelialization rates would be impacted by circulating EPC numbers. Additionally, the number of EPCs necessary to completely cover a device is miniscule in comparison to the number in the circulating pool. Statin therapy has been shown to increase circulating EPCs in patients with coronary artery disease (CAD) or CAD risk factors [46, 47], which may be used prior to device implantation.

## Conclusion

A universal, oriented antibody immobilization method has been established, that has the potential for clinical applications, such as modifying ePTFE graft material to mitigate its thrombotic propensity and possibly provide for improved long-term patency for small-diameter vascular grafts.

## Materials and methods

### Materials

Electropolished CC discs, CC stents, ePTFE grafts, and anti-human CD34 antibodies (mouse IgG2a) were provided by OrbusNeich Medical Technologies (Fort Lauderdale, FL, USA). Mouse isotype control (cat#: 015-000-002, 5 mg/ml) for CD34 antibody was obtained from Jackson ImmunoResearch Laboratories Inc. (West Grove, PA, USA). Mouse anti-pig CD31 antibody (cat#: MCA1746F) was purchased from Bio-Rad Canada (Mississauga, ON, Canada). FITC-conjugated rabbit anti human CD34 polyclonal antibody (cat#: 10,665-284) was provided by VWR Canada (Mississauga, ON, Canada). Recombinant human CD34 peptide containing the entire extracellular domain was procured from Abcam Inc., Toronto, ON, Canada (cat#: ab126924). Amino-(PEG)_8_-hydrazide-t-boc (the linker) was obtained from Quanta Biodesign (Plain City, OH, USA) and all other chemicals from Sigma-Aldrich Canada (Oakville, ON, Canada). CD34 + KG1a human leukemic progenitor cells, HEK293 human kidney epithelial cells, human umbilical vein endothelial cells (HUVECs), Iscove’s Modified Dulbecco's Medium (IMDM), Eagle's Minimum Essential Medium (EMEM), Endothelial Basal Medium (EBM) and fetal bovine serum (FBS) were purchased from ATCC (Gaithersburg, MD, USA). PD-10 Desalting Columns were from Fisher Scientific Canada (Ottawa, ON, Canada). The fluorescence dyes Calcein-AM, CMTMR and Sytox Green were obtained from Thermo Fisher Scientific Canada (Mississauga, ON, Canada).

### Oriented CD34 antibody coating

Oriented CD34 antibody or isotype coating, intermediate coating, and CD34 antibody coating without t-boc removal were done as follows.

#### Poly-dopamine base and PEG linker construction

The material to be coated was bath sonicated in ethanol, acetone and ddH_2_O for 10 min each. For the deposition of poly-dopamine base and PEG linker, the cleaned material was immersed in a mixture of equal volumes of 4 mg/ml dopamine hydrochloride and 50 mg/ml Amino-(PEG)_8_-hydrazide-t-boc solution, both prepared with 10 mM Tris.HCl (pH 8.5), and incubated by shaking at 250 rpm at 37 °C for 24 h. This led to the generation of substrates with intermediate coating only. After 3 washes with ddH_2_O and 3 washes with ethanol, 10 min each by shaking at 100 rpm at room temperature, the substrates with intermediate coating were either stored in the storage buffer (SB), i.e., phosphate buffered saline (PBS, pH 7.4) containing 100 units/ml of penicillin, 100 µg/mL of streptomycin and 2.5 µg/mL of amphotericin B, in a 4 °C refrigerator until further analyzed or processed further as described below.

#### Removal of t-boc

The substrate with intermediate coating was dipped in dichloromethane and incubated at room temperature for 10 min followed by incubation in 2 mg/ml iodine solution prepared with dichloromethane by shaking at 100 rpm at room temperature for 5 h.

#### Antibody oxidization

10 mM sodium periodate solution prepared with the oxidation buffer (20 mM sodium acetate and 15 mM sodium chloride, pH 5.6) was used for antibody oxidization. CD34 antibody or isotype control was mixed with 10 mM sodium periodate solution to reach a final concentration of 10 µg/ml. The mixture was then incubated (protected from light) by shaking at 100 rpm at room temperature for 30 min. Afterwards, sodium periodate was removed using the PD-10 Desalting Column according to the manufacturer’s instructions, and CD34 antibodies were eluted with oxidation buffer.

#### Oriented antibody immobilization

Following t-boc removal described above, the substrate was washed 3 times with dichloromethane, 3 times with ethanol and 3 times with ddH_2_O (10 min each time) by shaking at 100 rpm at room temperature. Finally, the substrate was immersed in the oxidized CD34 antibodies or isotype control, and incubated at 4 °C for 24 h. After incubation, the coated material was washed one time with PBS and stored in the SB in a 4 °C refrigerator until further analyzed.

#### Antibody coating without t-boc removal

The substrate with intermediate coating without t-boc removal was incubated directly with oxidized CD34 antibodies at 4 °C for 24 h. After one wash with PBS, the coated substrate was stored in the SB in a 4 °C refrigerator until further analyzed.

### Coating surface characterization

#### Scanning electron microscopy (SEM)

SEM was performed as described elsewhere [48].

#### Immunofluorescence analysis

CD34 antibodies coated on CC discs were evaluated by immunofluorescence analysis with isotype coated discs serving as control. Coated discs were placed in a 96-well plate (1 disc/well) and blocked with PBS containing 2% bovine serum albumin (BSA) at room temperature for 1 h. After 3 washes with PBS, the disc was incubated with 100 µl of CD34 peptide (5 µg/ml) prepared with the blocking buffer at room temperature for 2 h followed by 3 washes with PBS. Subsequently, the disc was incubated with FITC-conjugated anti-CD34 polyclonal antibodies (1:50 diluted with the blocking buffer) in dark at room temperature for 1 h. After 3 washes with PBS, the disc was mounted on a glass slide and observed under fluorescence microscope.

#### Mechanical characterization

The integrity and deformability of the coating was assessed on a Graftmaster RX coronary stent system (Abbott Vascular, Santa Clara, California). The Graftmaster stent is configured with a layer of ePTFE sandwiched between two identical stainless steel stents. The device was coated in its unexpanded state, then expanded to its fully deployed size at nominal expansion pressure, with a size matched compliant angioplasty balloon. The expanded stent system was then analyzed by SEM with a coated unexpanded coronary stent system serving as control.

#### X-ray photoelectron spectroscopy (XPS)

XPS was performed as described elsewhere [48].

#### Contact angle measurement

Contact angle measurement was performed for bare and coated ePTFE grafts using an optical NRL Model 100–00 contact angle goniometer (NRL C.A. goniometer, Ramé-Hart, Inc., Mountain Lakes, NJ, USA) as described elsewhere [25].

#### Cell growth analysis

The in vitro cell compatibility of the CD34 antibody coating was tested by cell growth on coated CC discs using HUVECs maintained in EBM containing 5% FBS. A coated disc was placed in a well of a 6-well plate followed by addition of 1 × 10^5^ HUVECs in 2 ml culture medium. The plate was transferred into a cell culture incubator. At 24 and 48 h after culture, the fluorescence dye Calcein-AM that only stains live cells was added to cells (final concentration: 1 µM) and cells were kept in the incubator for 30 min. Afterwards, the disc was removed, placed on a coverslip, and stained cells were watched and counted under fluorescence microscope. The cell number from 5 randomly chosen fields (20x) for each disc was calculated, and the average cell numbers were compared between different time points.

### In vitro cell capture assay

CD34 + KG1a cells were maintained in IMDM supplemented with 20% FBS. CD34- HEK293 cells were maintained in EMEM supplemented with 10% FBS. The ability of CD34 antibody-coated substrates to bind CD34 + cells was tested with mixed CD34 + KG1a and CD34- HEK293 cells that were pre-stained with different fluorescence dyes. Briefly, KG1a and HEK293 cells were stained with Calcein-AM and CMTMR respectively, both at a final concentration of 1 µM, according to the manufacturer’s instructions. Cells were then harvested, washed once with PBS and re-suspended in PBS at a density of 2 × 10^6^/ml for cell binding assay. The coated material was blocked with PBS containing 2% BSA at room temperature for 1 h followed by incubation with a mixture of equal volumes of KG1a and HEK293 cells in a 2 ml cryotube protected from light by shaking at 100 rpm at room temperature for 1 h. Subsequently, the substrate was thoroughly washed 6 times with PBS to remove non-specifically attached cells and bound cells were visualized under fluorescence microscope. Additionally, cell binding testing was done for coated substrates using KG1a cells with HEK293 cells serving as control. KG1a cells or HEK293 cells detached by trypsinization were washed once with PBS and re-suspended in PBS at a density of 10^6^ cells/ml. After blocking as described above, the coated substrate was incubated with either KG1a cells or HEK293 cells by shaking at 100 rpm at room temperature for 1 h. Subsequently, the material was thoroughly washed 6 times with PBS to remove non-specifically attached cells. After fixation in 4% paraformaldehyde prepared with PBS for 15 min, bound cells were stained with Sytox Green and visualized under fluorescence microscope. To test the stability of coated CD34 antibody, the coated material was stored in the SB in a 4 °C refrigerator for 2 weeks, and then tested by cell binding assays as described above.

### Pig carotid artery graft interposition

The animal protocol was approved by the Animal Care Committee of St. Michael’s Hospital, Unity Health Toronto, University of Toronto (approval number: ACC893), in accordance with the NIH Guide for the Care and Use of Laboratory Animals, 8th edition. CD34 antibody coated ePTFE grafts (5 mm in diameter) were tested for enhanced endothelialization in a carotid artery graft interposition model in a total of 9 Yorkshire male pigs weighing approximately 40 kg. Three pigs were used for each of the following grafts: CD34 antibody-coated, bare, and intermediately-coated. Before implantation, the graft was blocked in pig serum at room temperature for 30 min. Animals were fasted ~ 12 h and then anesthetized. A cervical incision was made after disinfection with alcohol and betadine to expose the unilateral carotid artery. A carotid artery interposition with the graft was performed as follows: intravenous unfractionated heparin was given and the carotid artery was clamped proximal and distal to the interposition site, and a segment of approximately 3 cm was excised and the ends of the artery were beveled. The graft was also beveled and trimmed to a length of 3 cm. Proximal and distal anastomoses were completed with a 7-0 polypropylene along a continuous suture line. Flow was restored by removing the clamps, and absence of proximal and distal anastomotic suture line leak confirmed. The cervical incision was closed in layers and the animals were allowed to recover and analgesic measures were taken by intramuscular administration of Anafen once (3 mg/kg) and oral administration of Metacam for 48 h (0.1 mg/kg). Each pig was orally administered 0.5 g of acetylsalicylic acid daily until the end-point study. At day 14 post surgery, pigs were anesthetized, and carotid ultrasound was performed using a handheld Doppler probe immediately before vessel exposure and explantation of the prosthetic material in order to confirm vessel patency. No carotid Doppler tracings were recorded. The grafts were explanted and gently flushed with PBS. Each graft was cut in two halves: one half for SEM analysis and the other half for immunostaining.

### Analysis of explanted grafts

#### SEM analysis

One half of each explanted graft was fixed with formaldehyde and glutaraldehyde 2.5% each in 0.1 M sodium cacodylate buffer (pH 7.2) at 4 °C for 24 h. The fixative was removed by washing grafts 3 times 10 min each in 0.1 M Sorensen’s Phosphate Buffer (pH 7.2). The graft was then fixed in 1% osmium tetroxide prepared with 0.1 M Sorensen’s Phosphate Buffer for 1.5 h and then washed 3 times 10 min each in 0.1 M Sorensen’s Phosphate Buffer. Afterwards, the graft was dehydrated through an ascending ethanol series of 50–100% ethanol solutions for 10 min each followed by infiltration with an ethanol:hexamethyldisilizane (HMDS) series at a ratio of 3:1, 1:1, 1:3 and 100% HMDS, all for 10 min each. After being left to dry overnight in 100% HMDS, the material was mounted on the SEM stub, sputter coated with platinum using the Bal-Tec SCD 050, and examined with a Hitachi S-2500 scanning electron microscope. The endothelium covered areas were determined in a blind manner using the Image J software as follows: the area covered or uncovered with endothelial cells that displayed a cobblestone morphology was manually selected using the free hand selection tool, and the selected area was then automatically calculated by the “Analyze > Measure” command with the “Area” being selected from the “Results > Set Measurement” menu. Each half graft was divided into 3 longitudinal sections covered by 3 consecutive SEM images (approximately 5 mm/section) for measurement, and the sum of covered or uncovered areas from the 3 sections were used to calculate the percentage of endothelialization as follows: sum of endothelialization area/(sum of endothelialization area + sum of non-endothelialization area) × 100%.

#### Immunostaining

The explanted grafts (one half of each graft) were fixed with 4% paraformaldehyde at room temperature for 15 min, blocked in PBS containing 2% BSA for 1 h and incubated with the mouse anti-pig CD31 antibody (1:100 dilution in PBS containing 2% BSA) at room temperature for 1 h. Following 3 washes with PBS, 5 min each, the grafts were incubated with the secondary antibodies (1:1000 dilution, DAPI for nuclear staining) under the light protection for 1 h. After 3 washes with PBS 5 min each, the graft was mounted on a coverslip and visualized under confocal microscope.

### Statistical analysis

All data from in vitro study of bare and coated substrates were analyzed with Student t test. Endothelium covered areas in explanted grafts in animal study were analyzed with ANOVA with post-hoc Tukey test. A two-tailed *p* < 0.05 was considered statistically significant.

## Supplementary Information

**Additional file 1:** **Supplementary Figure 1. **Immunofluorescence analysis of immobilized CD34 antibody. The CC disc coated with CD34 antibody or the isotype control was incubated with human CD34 peptide, and the bound CD34 peptide, if any, was detected with FITC-conjugated anti human CD34 polyclonal antibody. As shown in this figure, the fluorescence dye FITCwas detectable on the CC disc coated with CD34 antibodybut not the isotype.

**Additional file 2:** **Supplementary Figure 2. **XPS spectra. Representative XPS spectra of bare and coated PTFE material, and of a bare and a coated disc are shown in this figure. Fluorine signal in the ePTFE material was significantly reduced after coating, and signals of cobalt and chromium were absent in the coated disc. Co: cobalt; Cr: chromium; F: fluorine; C: carbon; O: oxygen; and N: nitrogen.

**Additional file 3:** **Supplementary Figure 3. **KG1a cell binding analysis of substrates coated with different methods. As shown in this figure, only the substrate fully coated with CD34 antibody bound CD34+ KG1a cells, while the isotype coated substrate, intermediately coated substrate or the substrate coated with CD34 antibody without-boc removal did not bind KG1a cells.

## Data Availability

All data and materials supporting the conclusions of this study are presented in the main manuscript and the supplementary file.
