# Supplementary figures and images for: Development of a universal, oriented antibody immobilization method to functionalize vascular prostheses for enhanced endothelialization for potential clinical application
Source: J Biol Eng. 2023 Jun 1;17:37. doi: 10.1186/s13036-023-00356-6 (PMC10236874; doi:10.1186/s13036-023-00356-6)

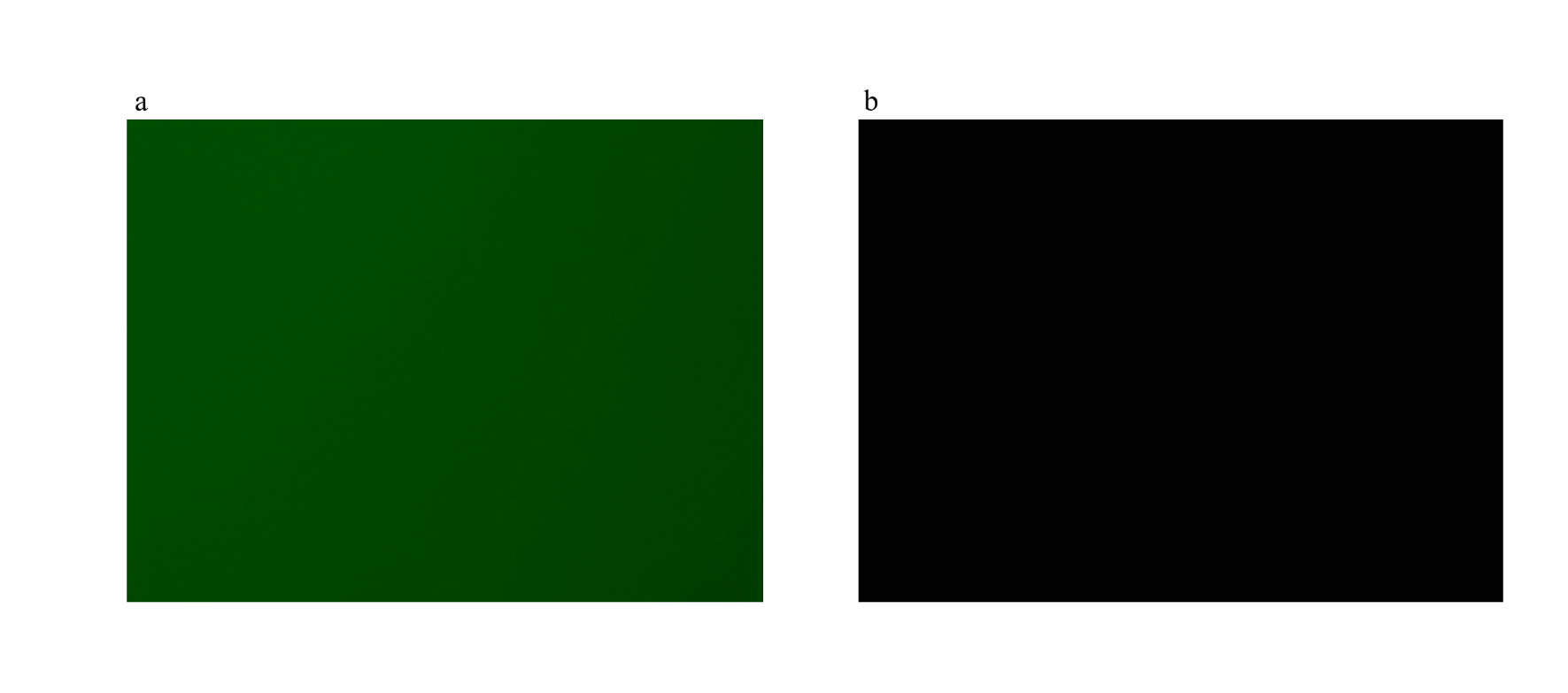

Supplement: Supplementary file 1 — Additional file 1: Supplementary Figure 1. Immunofluorescence analysis of immobilized CD34 antibody. The CC disc coated with CD34 antibody or the isotype control was incubated with human CD34 peptide, and the bound CD34 peptide, if any, was detected with FITC-conjugated anti human CD34 polyclonal antibody. As shown in this figure, the fluorescence dye FITCwas detectable on the CC disc coated with CD34 antibodybut not the isotype. [file 13036_2023_356_MOESM1_ESM.docx]

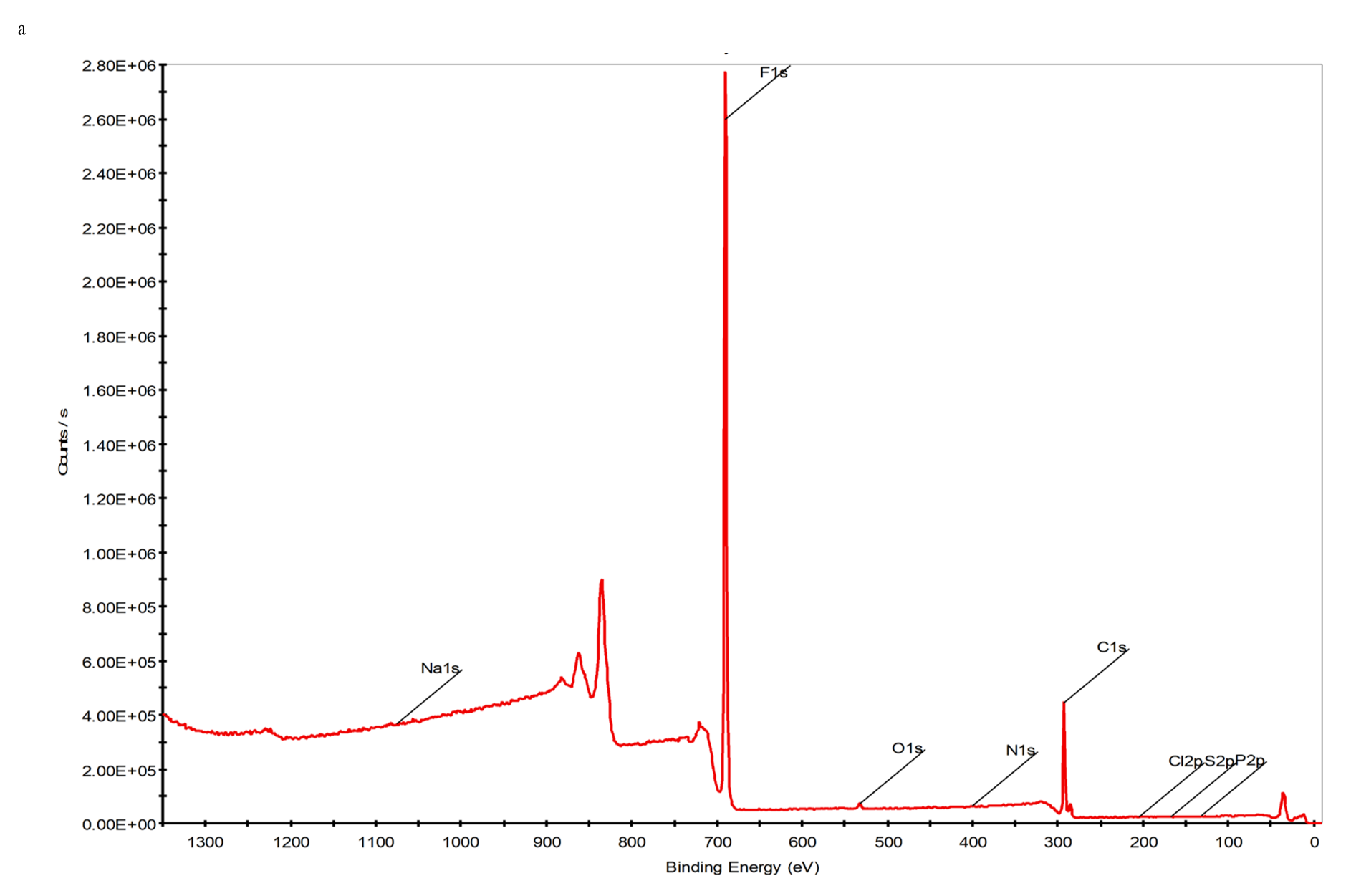


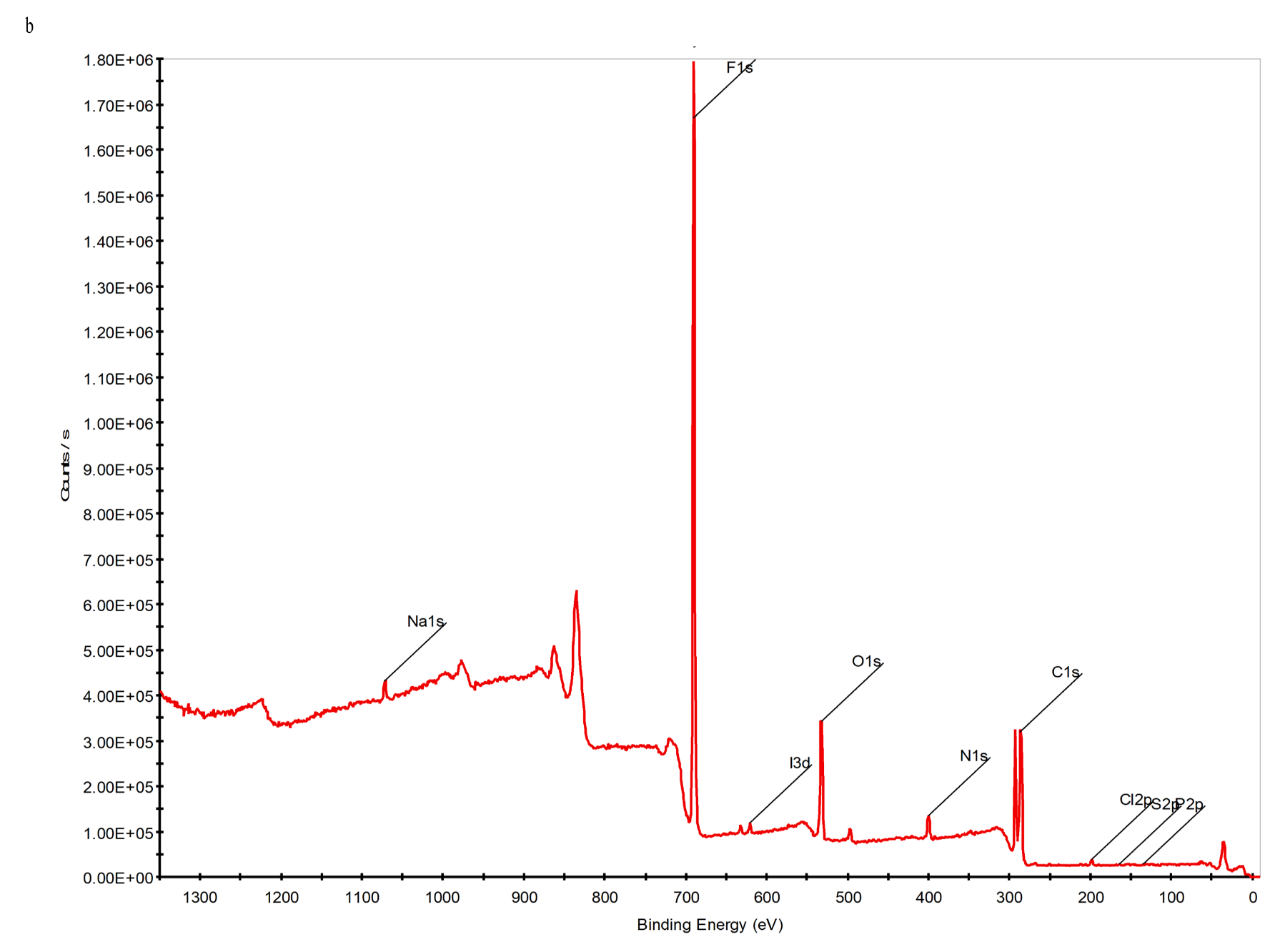


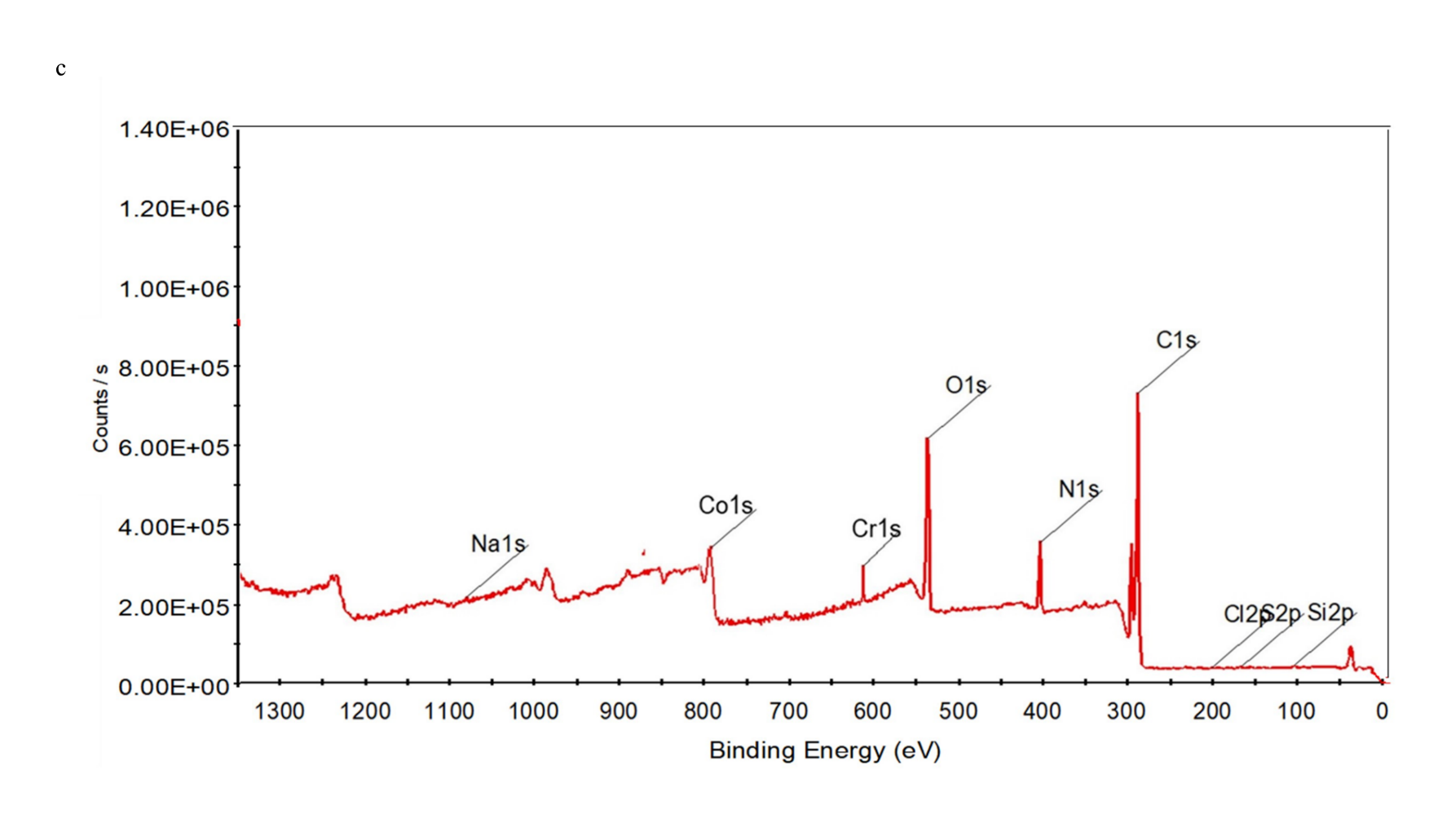


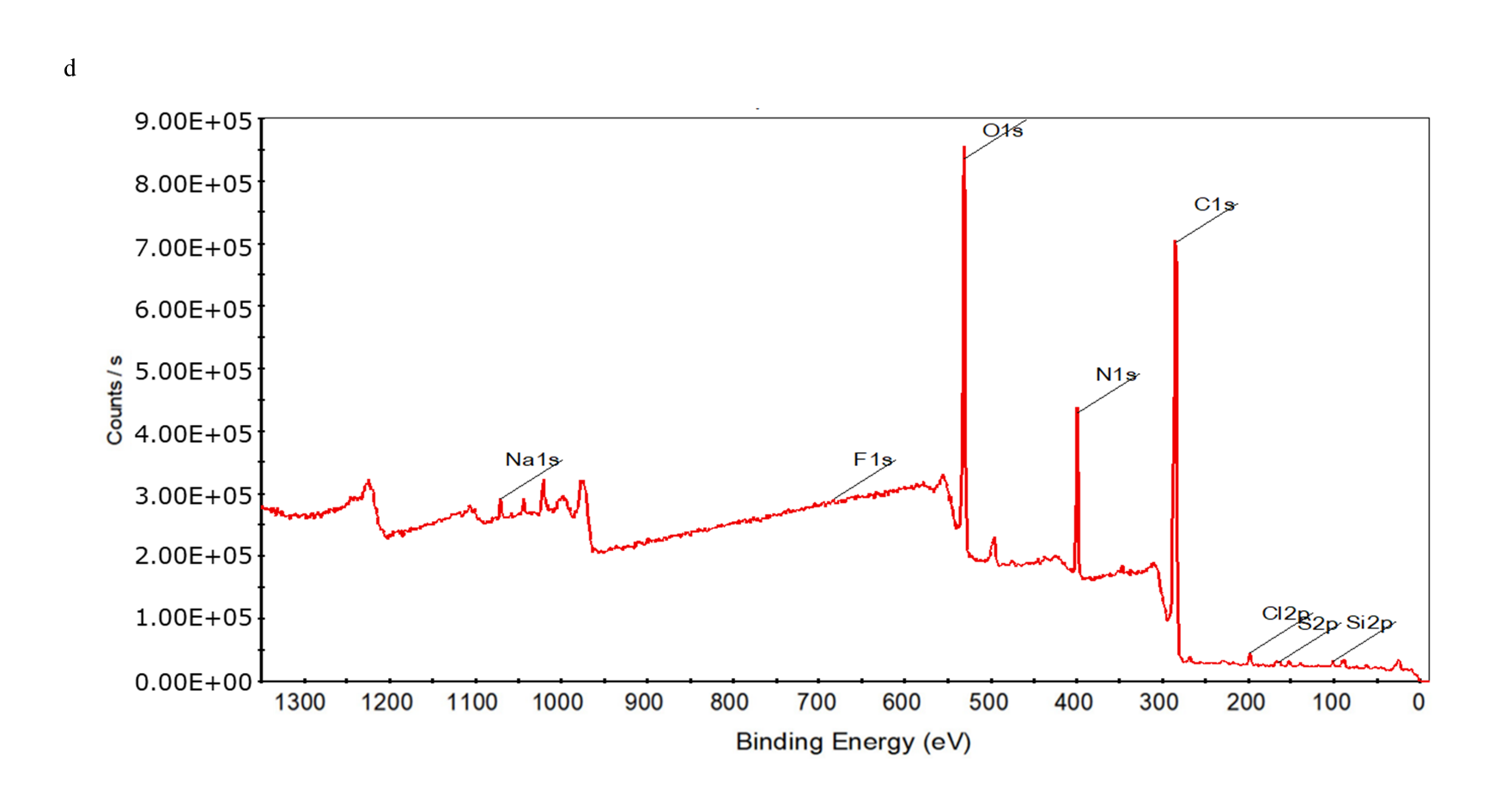

Supplement: Supplementary file 2 — Additional file 2: Supplementary Figure 2. XPS spectra. Representative XPS spectra of bare and coated PTFE material, and of a bare and a coated disc are shown in this figure. Fluorine signal in the ePTFE material was significantly reduced after coating, and signals of cobalt and chromium were absent in the coated disc. Co: cobalt; Cr: chromium; F: fluorine; C: carbon; O: oxygen; and N: nitrogen. [file 13036_2023_356_MOESM2_ESM.docx]

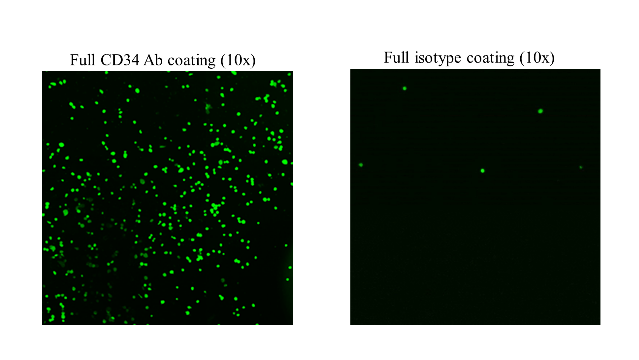


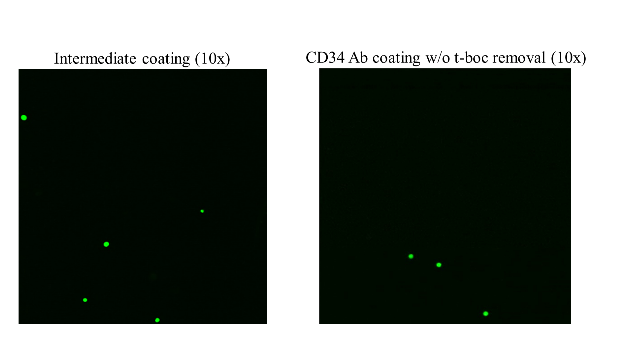

Supplement: Supplementary file 3 — Additional file 3: Supplementary Figure 3. KG1a cell binding analysis of substrates coated with different methods. As shown in this figure, only the substrate fully coated with CD34 antibody bound CD34+ KG1a cells, while the isotype coated substrate, intermediately coated substrate or the substrate coated with CD34 antibody without-boc removal did not bind KG1a cells. [file 13036_2023_356_MOESM3_ESM.docx]
